# Endoscopic Band Ligation for Gastric Antral Vascular Ectasia: Report of Two Cases at a Mexican Tertiary Care Center

**DOI:** 10.7759/cureus.114681

**Published:** 2026-08-17

**Authors:** Anel Y Avila-Franco, Katia D López-García, Yoeli M Escandón-Espinoza

**Affiliations:** 1 Gastrointestinal Endoscopy, Hospital Regional de Alta Especialidad Bicentenario de la Independencia, Tultitlán, MEX

**Keywords:** argon plasma coagulation, case report, endoscopic band ligation, gastric antral vascular ectasia, gastrointestinal bleeding

## Abstract

Gastric antral vascular ectasia (GAVE) is an uncommon yet clinically significant cause of chronic upper gastrointestinal bleeding, frequently associated with chronic anemia and recurrent transfusion requirements. Although argon plasma coagulation (APC) remains the most widely used endoscopic therapy, endoscopic band ligation (EBL) is a safe and effective alternative for the treatment of GAVE. We report two female patients with distinct clinical backgrounds and endoscopic presentations of GAVE who underwent EBL at a Mexican tertiary care center where APC was temporarily unavailable. One patient had rheumatoid arthritis and presented with melena and severe anemia, while the other had liver cirrhosis with a history of esophageal varices and presented with chronic anemia and melena. Both patients experienced clinical and hematologic improvement following EBL, with no procedure-related complications. Endoscopic follow-up demonstrated marked regression of GAVE lesions, with one patient requiring a second session because of residual lesions. These findings add to the growing body of evidence supporting EBL as a safe and effective therapeutic option for GAVE across different clinical presentations.

## Introduction

Gastric antral vascular ectasia (GAVE) is an uncommon cause of non-variceal upper gastrointestinal bleeding, accounting for approximately 4% of cases. It is characterized by dilated vascular lesions in the gastric antrum, presenting as longitudinal erythematous stripes or diffuse punctate lesions [1,2]. Clinically, GAVE commonly manifests as chronic occult bleeding leading to iron-deficiency anemia and recurrent transfusion requirements [3,4]. GAVE is associated with systemic diseases, particularly liver cirrhosis, autoimmune disorders, and metabolic conditions [2,3,5]. Its pathophysiology remains incompletely understood and is likely multifactorial, involving abnormal antral motility, mechanical stress, and dysregulation of vasoactive substances [3,6].

Endoscopic therapy is the mainstay of treatment. Argon plasma coagulation (APC) has been the most widely used modality because of its availability and favorable safety profile. However, its effectiveness may be limited by its superficial depth of coagulation, often requiring multiple treatment sessions and demonstrating variable long-term outcomes with significant recurrence rates [7,8]. Endoscopic band ligation (EBL) is a safe and effective alternative that achieves mechanical obliteration of mucosal and submucosal vessels. Comparative studies and meta-analyses have demonstrated that EBL may achieve higher eradication rates, require fewer treatment sessions, reduce transfusion requirements, and maintain a comparable safety profile [9,10]. Although APC remains the standard and most widely used therapy, access to argon gas may be limited in some centers, making alternative endoscopic therapies valuable in selected clinical settings.

Given the heterogeneity in clinical presentation, underlying pathophysiology, and response to treatment, GAVE remains a challenging condition to manage. We describe two cases of GAVE with different endoscopic patterns and underlying clinical backgrounds managed with endoscopic band ligation, illustrating its applicability and safety across different clinical presentations.

## Case presentation

Case 1

A 67-year-old woman with a history of rheumatoid arthritis treated with adalimumab presented with a three-month history of progressive fatigue, generalized weakness, exertional dyspnea, and intermittent melena. She denied recent use of nonsteroidal anti-inflammatory drugs, corticosteroids, anticoagulants, or antiplatelet agents, as well as hematemesis, abdominal pain, weight loss, or previous episodes of gastrointestinal bleeding.

On admission, she was hemodynamically stable. Physical examination revealed marked conjunctival pallor. The remainder of the examination was unremarkable. Laboratory evaluation demonstrated severe microcytic iron-deficiency anemia, with a hemoglobin level of 6.7 g/dL, mean corpuscular volume of 68 fL, ferritin of 16 ng/mL, serum iron of 29 μg/dL, total iron-binding capacity of 408 μg/dL, and transferrin saturation of 9%, confirming iron deficiency. Platelet count, renal function, liver function tests, and coagulation profile were within normal limits. Because of symptomatic severe anemia, she received one unit of packed red blood cells. Following endoscopic treatment, intravenous iron supplementation was administered before discharge, and oral proton pump inhibitor therapy was prescribed.

**Figure 1:**
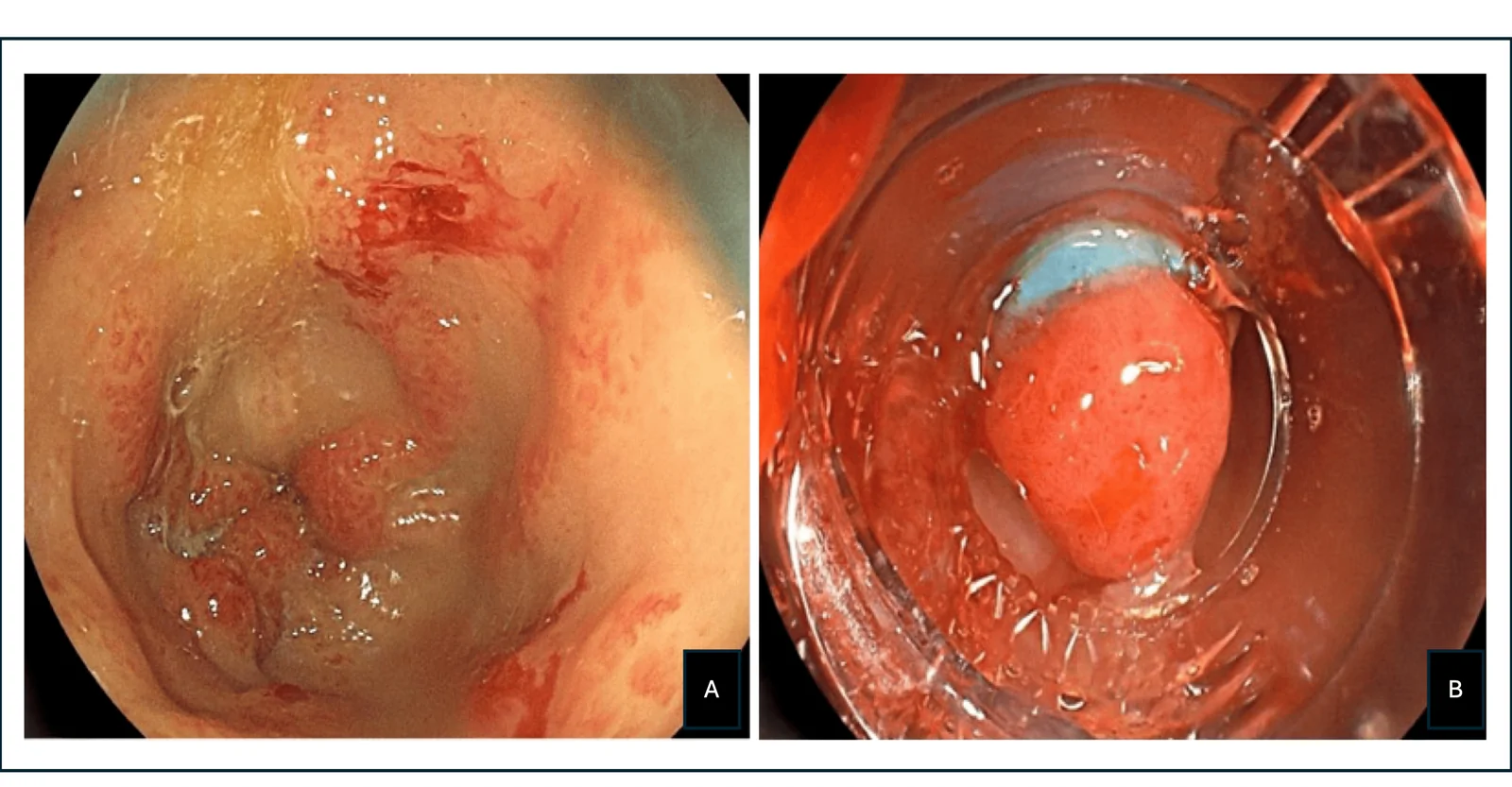
Initial Endoscopy and Endoscopic Band Ligation (A) Classic striped gastric antral vascular ectasia (GAVE). (B) Endoscopic band ligation of the affected mucosa using a multiband ligation device.

Esophagogastroduodenoscopy (EGD) revealed multiple longitudinal erythematous vascular stripes confined to the gastric antrum, consistent with the classic striped pattern of GAVE (Figure 1A). No other potential sources of upper gastrointestinal bleeding were identified. EBL was performed using a multiband ligation device (Speedband Superview Super 7; Boston Scientific, Marlborough, MA, USA). Five bands were deployed over the affected mucosa (Figure 1B). The procedure was completed without immediate complications. 

**Figure 2:**
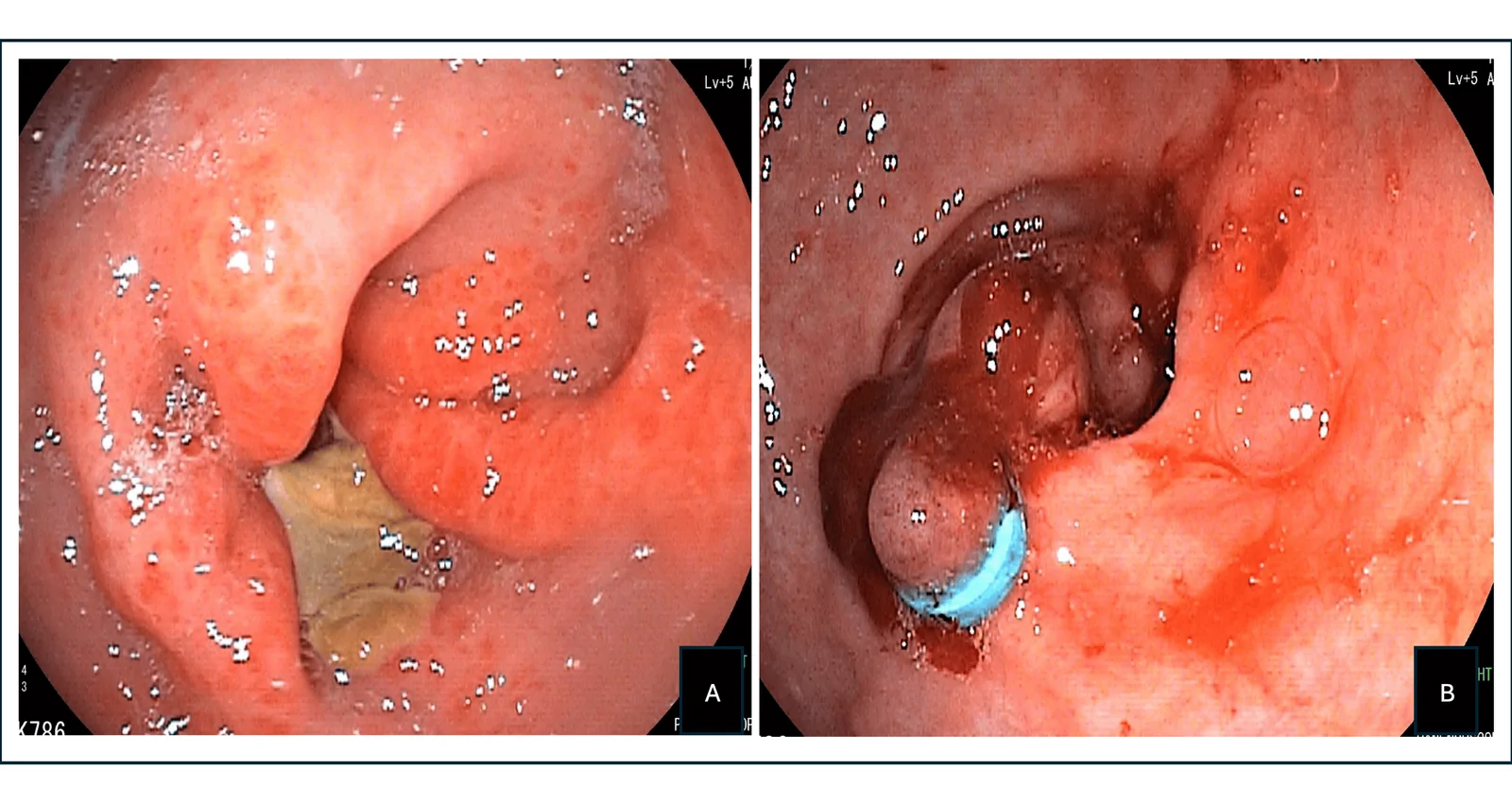
Follow-Up Endoscopy and Second Endoscopic Band Ligation (A) Follow-up endoscopy after the first endoscopic band ligation session showing marked reduction of gastric antral vascular ectasia (GAVE), post-banding ulcers, and residual ectatic areas. (B) Second endoscopic band ligation session.

At four-week follow-up, the patient reported complete resolution of melena and significant improvement in fatigue and exertional dyspnea. Hemoglobin increased to 11.1 g/dL. Follow-up EGD demonstrated marked regression of the vascular lesions, although residual ectatic areas and post-banding ulcers remained (Figure 2A). A second EBL session was performed using three additional bands (Figure 2B) without complications.

**Figure 3:**
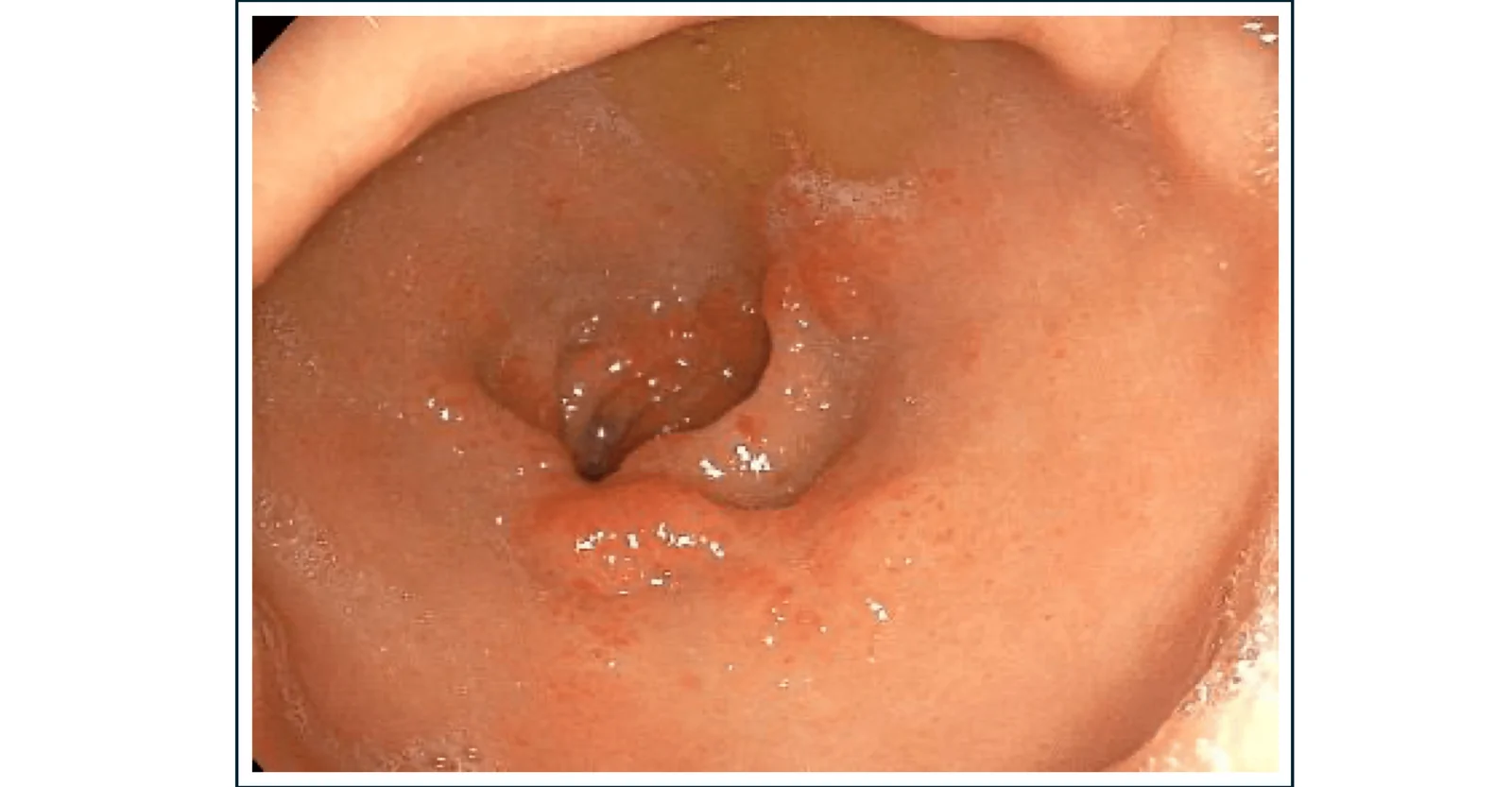
Final Endoscopic Follow-Up Follow-up endoscopy after the second endoscopic band ligation session demonstrating sustained regression of gastric antral vascular ectasia (GAVE).

Six weeks after the second EBL session, follow-up endoscopy demonstrated sustained regression of the GAVE lesions (Figure 3). As no significant residual lesions requiring treatment were identified, no additional endoscopic therapy was performed. The patient has remained under clinical follow-up without recurrent gastrointestinal bleeding.

Case 2

A 56-year-old woman with liver cirrhosis secondary to metabolic dysfunction-associated steatotic liver disease (MASLD), receiving propranolol, lactulose, and rifaximin, and with a history of previously eradicated esophageal varices, presented with a several-month history of chronic fatigue, progressive weakness, and intermittent melena. She denied hematemesis, abdominal pain, weight loss, and recent use of nonsteroidal anti-inflammatory drugs, anticoagulants, or antiplatelet agents.

On admission, she was hemodynamically stable. Physical examination revealed conjunctival pallor. The abdomen was soft and non-tender, with no clinical evidence of ascites or signs of peritoneal irritation. No jaundice or hepatic encephalopathy was observed. Laboratory evaluation demonstrated normocytic normochromic anemia, with a hemoglobin level of 8.3 g/dL. Liver function tests were consistent with underlying chronic liver disease without evidence of acute hepatic decompensation. Given her hemodynamic stability and hemoglobin level, neither blood transfusion nor iron supplementation was required before endoscopic evaluation.

**Figure 4:**
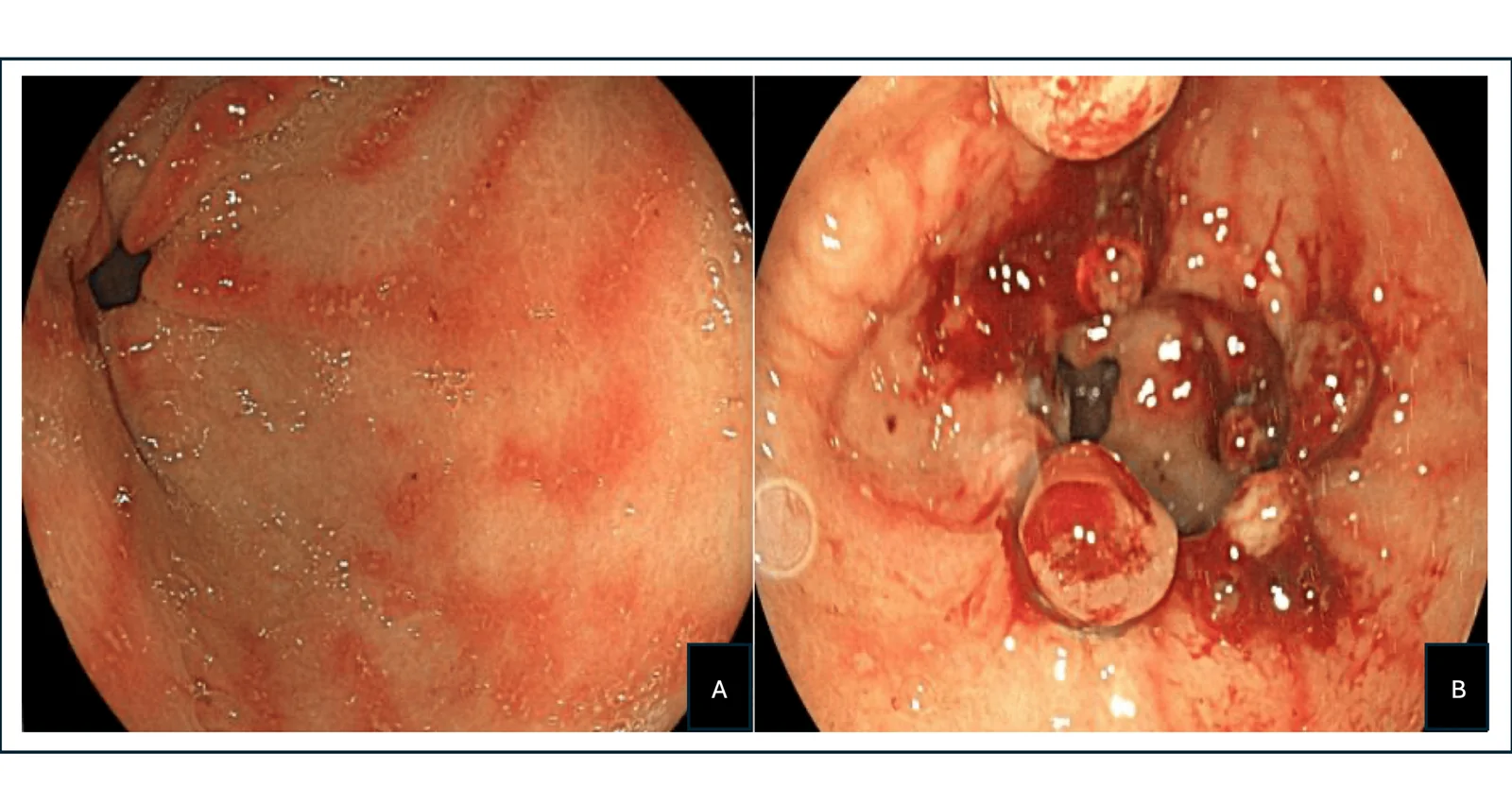
Initial Endoscopy and Endoscopic Band Ligation (A) Diffuse gastric antral vascular ectasia (GAVE). (B) Endoscopic band ligation of the ectatic vascular lesions

EGD revealed diffuse punctate vascular ectasias confined to the gastric antrum, consistent with the diffuse pattern of GAVE (Figure 4A). Mild portal hypertensive gastropathy was observed in the gastric body, while previously eradicated esophageal varices showed no evidence of recurrence. No other potential sources of upper gastrointestinal bleeding were identified. EBL was performed using the same multiband ligation device (Speedband Superview Super 7). Seven bands were deployed over the affected mucosa (Figure 4B). The procedure was completed without immediate complications, and oral proton pump inhibitor therapy was prescribed following EBL.

**Figure 5:**
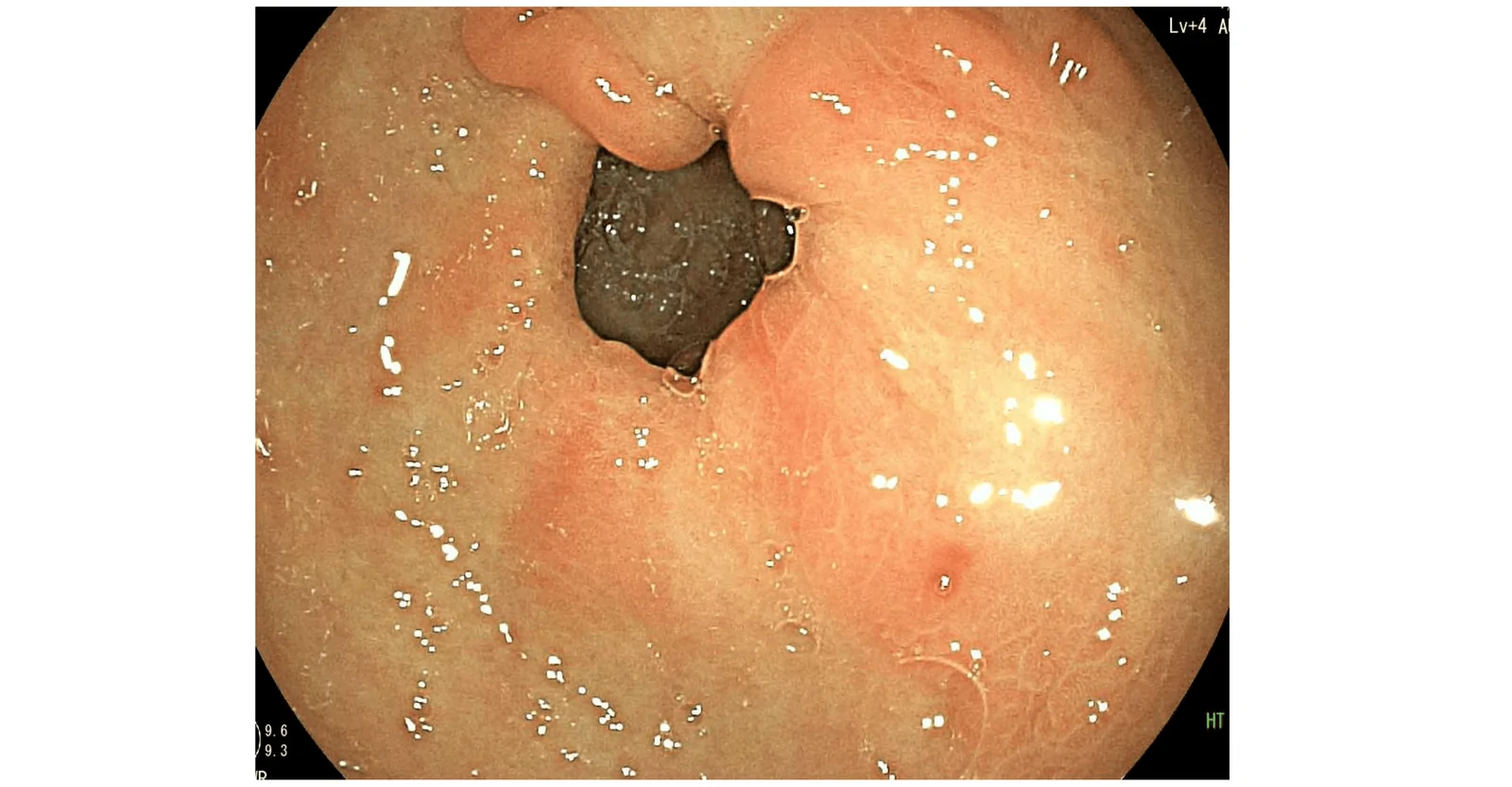
Follow-Up Endoscopy After Endoscopic Band Ligation Follow-up endoscopy demonstrating marked regression of diffuse gastric antral vascular ectasia (GAVE) after endoscopic band ligation.

Four weeks after treatment, melena had resolved, and hemoglobin increased to 9.5 g/dL. Follow-up EGD demonstrated marked regression of the vascular ectasias, with post-banding mucosal scarring and no evidence of active bleeding (Figure 5). As no significant residual lesions requiring treatment were identified, no additional endoscopic therapy was performed. The patient has remained under clinical follow-up for six months without recurrent episodes of gastrointestinal bleeding.

## Discussion

GAVE encompasses distinct endoscopic patterns and clinical associations and remains an important cause of chronic upper gastrointestinal bleeding [11,12]. Diffuse punctate lesions are more frequently observed in patients with cirrhosis, while the striped pattern is more characteristic of non-cirrhotic patients [13], as observed in our two cases.

APC remains the standard endoscopic treatment for GAVE, although its superficial coagulation, need for repeated sessions, and recurrence are important limitations [8,14,15]. EBL offers a mechanical approach to vascular obliteration and has shown favorable efficacy and safety outcomes in previous studies [9,10]. These findings have led to increasing recognition of EBL as an effective therapeutic option in clinical practice.

Our cases further illustrate the clinical heterogeneity of GAVE. Although both patients presented with chronic gastrointestinal bleeding secondary to GAVE, they differed substantially in their underlying diseases, hematologic profiles, and endoscopic phenotypes. The patient with rheumatoid arthritis presented with classic striped GAVE and iron-deficiency anemia, whereas the patient with MASLD-related cirrhosis exhibited diffuse GAVE, concomitant mild portal hypertensive gastropathy, and normocytic normochromic anemia, likely reflecting the multifactorial nature of anemia in chronic liver disease.

Our second patient illustrates the coexistence of GAVE and mild portal hypertensive gastropathy, a recognized but clinically important scenario in patients with cirrhosis. Although both entities may present with chronic gastrointestinal bleeding, they differ in their endoscopic appearance, pathophysiology, and therapeutic approach. In this case, the vascular ectasias were confined to the gastric antrum, consistent with diffuse GAVE, whereas mild portal hypertensive gastropathy was limited to the gastric body. Because the endoscopic findings were characteristic of GAVE, EBL was selected as the endoscopic treatment.

At the time of treatment, APC was temporarily unavailable at our institution because of a shortage of argon gas. Given the available evidence supporting EBL for GAVE, both patients underwent EBL. Both patients experienced resolution of gastrointestinal bleeding, improvement in hemoglobin levels, and marked regression of the vascular lesions on follow-up endoscopy, without procedure-related complications. In the first patient, post-banding ulcers were observed as an expected consequence of EBL and healed without delayed bleeding or other adverse events, consistent with the favorable safety profile reported in previous studies [9,10]. This is particularly relevant in diffuse GAVE, which has historically been associated with more challenging management and higher recurrence rates [2].

This report has several limitations. The small sample size, short follow-up, and absence of a direct comparison with APC limit the generalizability of our findings. In addition, complete iron studies were unavailable for the second patient, limiting further characterization of the etiology of anemia. 

## Conclusions

EBL resulted in favorable clinical, hematologic, and endoscopic outcomes in two patients with GAVE. Our experience is consistent with the current evidence supporting EBL as an effective endoscopic treatment for GAVE and contributes additional real-world experience from a Mexican tertiary care center. The favorable outcomes observed in both cases further support the role of EBL as an effective therapeutic option across the spectrum of GAVE.
